# Generalized nodular tinea profunda in an immunosuppressed patient caused by *Trichophyton rubrum*^☆^

**DOI:** 10.1016/j.abd.2021.08.018

**Published:** 2023-04-22

**Authors:** Wei Li, Kun-E Lu, Sui-Qing Cai, Li-Min Lao

**Affiliations:** Department of Dermatology, The Second Affiliated Hospital, Zhejiang University School of Medicine, Hangzhou, China

Dear Editor,

We report the case of a 65-year-old man with generalized subcutaneous nodules with intense pruritus for 1 year (Fig. 1). He had a history of diabetes mellitus for 7 years, hypertension and chronic kidney disease for 5 years, and Bullous Pemphigoid (BP) for 2 years. Systemic glucocorticoid (oral prednisone 25 mg twice a day) was prescribed to treat his BP for more than 1 year.

**Figure 1:**
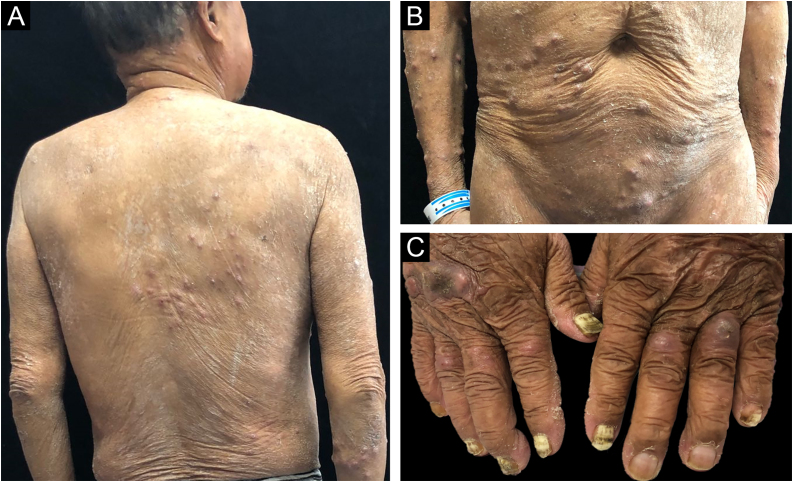
Clinical aspect: (A‒C) Multiple papules and subcutaneous nodules and onychomycosis.

After admission, blood test for fungal glucan was 213 pg/mL (the normal is lower than 60 pg/mL), which indicated a deep fungal infection. Blood culture for fungi was negative, and no lymphadenopathy was detected by ultrasonography. The light microscopy showed epidermal hyperplasia, dermal abscess, and infiltration of neutrophils, lymphocytes, epithelioid cells, and scattered multinucleated giant cells (Fig. 2A). Intracellular hyphae were observed in multinucleated cells in the granuloma (Fig. 2B). The skin sample was also sent for Next Generation Sequencing (NGS) to identify the pathogen. The NGS reported *Trichophyton rubrum* nucleotide sequences (Cover rate: 0.0199%) in DNA extracted from the skin specimen. Considering the patient’s clinical and histological manifestations, and the notable high sequencing reads compared to a negative control, we established the diagnosis of generalized nodular tinea profunda caused by *Trichophyton rubrum*. Oral therapy with 250 mg terbinafine per day was initiated and the nodules regressed completely after 3 months.

**Figure 2:**
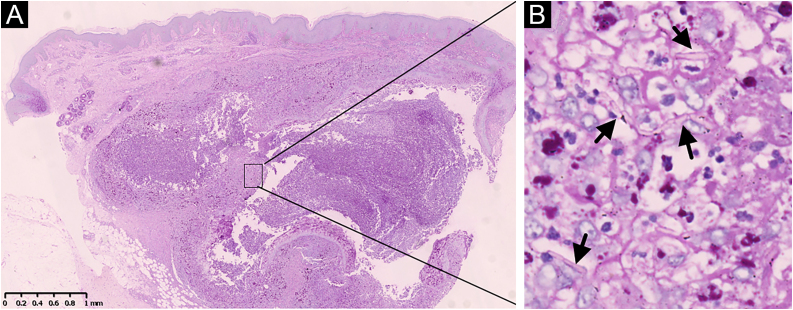
Light microscopy: (A) dermal abscess (periodic acid-Schiff, ×20); (B) Intracellular hyphae (arrow heads) in multinucleated cells in granuloma (periodic acid-Schiff, ×400).

*Trichophyton rubrum* often causes superficial dermatomycosis, such as tinea manus, tinea pedis and tinea corporis. But in very few cases, *T. rubrum* penetrate into the dermis and subcutaneous tissue, causing tinea profunda, also called deep dermatophytosis.^1^ Tinea profunda is characterized by the extension of dermatophyte infection beyond the perifollicular area, sometimes spreading to lymph nodes.^2^ Most tinea profunda patients have innate or acquired immunodeficiency, including malnutrition, diabetes, leukemia, lymphoma, Acquired Immunodeficiency Syndrome, solid organ transplantation, and chronic kidney disease.^2^

In the present case, diabetes mellitus, chronic kidney disease and systemic glucocorticoid treatment for Bullous Pemphigoid (BP) inhibited the patient’s innate and acquired immune system. Although the patient’s blood test was positive to fungal glucan, suggesting a deep fungal infection, the blood fungal culture was negative, and no lymphadenopathy was found by ultrasonography. The patient had onychomycosis for many years and did not received any treatment (Fig. 1C). As BP causes pruritus, the superficial dermatophyte may have been inoculated through the patient’s scratching, leading to generalized nodular tinea profunda.

## Financial Support

This study was funded by a grant from the 10.13039/501100001809National Natural Science Foundation of China (NFSC) (81874248).

## Authors’ contributions

Wei Li: Critical review of the literature; critical review of the manuscript.

Kun-E Lu: Drafting and editing of the manuscript.

Sui-Qing Cai: Design and planning of the study.

Li-Min Lao: Approval of the final version of the manuscript.

## Conflicts of interest

None declared.
